# The Changing Epidemiological Profile of HIV-1 Subtype B Epidemic in Ukraine

**DOI:** 10.1089/aid.2018.0167

**Published:** 2019-01-31

**Authors:** Tetyana I. Vasylyeva, Mariia Liulchuk, Louis du Plessis, Esther Fearnhill, Victoriia Zadorozhna, Nataliia Babii, Alla Scherbinska, Vladimir Novitsky, Oliver G. Pybus, Nuno R. Faria

**Affiliations:** ^1^ Department of Zoology, University of Oxford, Oxford, United Kingdom.; ^2^ L.V. Gromashevskij Institute of Epidemiology and Infectious Diseases of National Academy of Sciences of Ukraine, Kyiv, Ukraine.; ^3^ Institute for Global Health, University College London, United Kingdom.; ^4^ Department of Immunology and Infectious diseases, Harvard TH Chan School of Public Health, Boston, Massachusetts.

**Keywords:** HIV-1, B_FSU_, Ukraine, molecular epidemiology, people who inject drugs

## Abstract

While HIV-1 subtype B has caused a large epidemic in the Western world, its transmission in Ukraine remains poorly understood. We assessed the genetic diversity of HIV-1 subtype B viruses circulating in Ukraine, characterized the transmission group structure, and estimated key evolutionary and epidemiological parameters. We analyzed 120 HIV-1 subtype B *pol* sequences (including 46 newly generated) sampled from patients residing in 11 regions of Ukraine between 2002 and 2017. Phylogenies were estimated using maximum likelihood and Bayesian phylogenetic methods. A Bayesian molecular clock coalescent analysis was used to estimate effective population size dynamics and date the most recent common ancestors of identified clades. A phylodynamic birth–death model was used to estimate the effective reproductive number (*R_e_*) of these clades. We identified two phylogenetically distinct predominantly Ukrainian (≥75%) clades of HIV-1 subtype B. We found no significant transmission group structure for either clade, suggesting frequent mixing among transmission groups. The estimated dates of origin of both subtype B clades were around early 1970s, similar to the introduction of HIV-1 subtype A into Ukraine. *R_e_* was estimated to be 1.42 [95% highest posterior density (HPD) 1.26–1.56] for Clade 1 and 1.69 (95% HPD 1.49–1.84) for Clade 2. Evidently, the subtype B epidemic in the country is no longer concentrated in specific geographical regions or transmission groups. The study results highlight the necessity for strengthening preventive and monitoring efforts to reduce the further spread of HIV-1 subtype B.

## Introduction

Ukraine has one of the worst HIV epidemics among European countries: in 2015, the country had the highest rate of new HIV infections in Europe (∼30 cases per 100,000 people).^1^ In the 1990s, people who inject drugs (PWID) were the transmission group that accounted for majority of new HIV infections in Ukraine.^2,3^ However, since the late 2000s, transmission among heterosexual nondrug-injecting populations has prevailed.^4^

Even though HIV-1 subtype B and CRF03_AB (the latter first found in the Russian city Kaliningrad^5^) were circulating in the country since the 1990s, the majority of HIV-1 infections in Ukraine are caused by HIV-1 subtype A.^6,7^ In 2015, 89% of HIV-1 sequences in the Ukrainian HIV database, a drug resistance screening database containing sequences from HIV patients in Ukraine, were identified as HIV-1 subtype A.^8^ Similarly, 88% of Ukrainian sequences in the Los Alamos HIV sequence database are classified as HIV-1 subtype A.^9^ To date, most molecular epidemiology studies of the Ukrainian epidemic have focused on HIV-1 subtype A in PWID populations.^10–12^

HIV-1 subtype B, which is the most prevalent HIV subtype in most Western European countries, has limited distribution and prevalence in Ukraine compared with HIV-1 subtype A. In 2012–2015, 4% (19 of 448) of sequences in the Ukrainian Drug Resistance database were identified as HIV-1 subtype B,^8^ as were <10% of Ukrainian sequences in the Los Alamos HIV database.^9^ However, extrapolation of these rates (4%–10%) to the estimated 220,000 HIV-infected people in the country^1^ suggests that HIV-1 subtype B accounts for about 9,000–22,000 HIV infections in Ukraine.

The genetic diversity of HIV-1 subtype B in Ukraine seems to be characterized by two different cocirculating lineages: one is diagnosed mostly in PWID and characterized by very low genetic diversity, while another is more genetically similar to subtype B strains observed in Western European countries.^7,13^ The first lineage was not only reported mainly in Mykolaiv, a port city in the south of Ukraine, but was also observed in some other countries of the former Soviet Union (FSU) and was subsequently called B_FSU_.^7,14^ Two studies from Russia and Azerbaijan have shown that local HIV-1 subtype B infections among PWID clustered with viral sequences previously diagnosed in patients in Mykolaiv and Kyiv.^15,16^ A recent study demonstrated high levels of phylogenetic clustering (66%) among men who have sex with men (MSM) and heterosexual men infected with HIV-1 subtype B in Kyiv, the capital of Ukraine.^17^

The epidemiology of HIV-1 subtype B transmission across transmission groups, such as MSM and PWID, and among geographic regions in Ukraine remains poorly understood. In this study, we describe the dynamics of the HIV-1 subtype B epidemic in 11 Ukrainian regions between 2002 and 2017. We apply molecular epidemiological methods to estimate the dates of introduction of HIV-1 subtype B into Ukraine and assess the transmission dynamics of the most prevalent HIV-1 subtype B lineages.

## Materials and Methods

### Data

#### Genetic data

Ukrainian HIV-1 subtype B genetic sequences were derived from three sources. First, we report 46 new HIV-1 subtype B *pol* gene sequences from patients visiting Ukrainian AIDS centers (the hospitals for HIV treatment and care) in 2012–2017, which were made available through the Ukrainian HIV drug resistance database. These data were sequenced at the L.V. Gromashevskij Institute of Epidemiology and Infectious Diseases in Kyiv using the ViroSeq^®^ HIV-1 Genotyping System, v2.0, Kit (Celera Corporation, Abbott). These 46 sequences represent 11 administrative regions of Ukraine: Mykolaiv (*N* = 24), Kyiv (*N* = 6), Odessa (*N* = 3), Vinnytsya (*N* = 3), Dnipro (*N* = 2), Kharkiv (*N* = 2), Sumy (*N* = 2), and Ivano-Frankivsk, Kherson, Lugansk, and Zhytomyr (*N* = 1 each). Second, we used 73 HIV-1 subtype B *pol* sequences collected as part of the CASCADE (Concerted Action on SeroConversion to AIDS and Death in Europe) cohort study in Kyiv in 2013–2015.^17^ These data were collected anonymously in four infectious disease clinics that are part of the Kyiv AIDS Centre^18^ and were sequenced at University College London Hospital laboratories on Sanger or Illumina MiSeq platforms. Third, we accessed the Los Alamos HIV database^9^ and retrieved all available HIV-1 subtype B sequences (*N* = 9) collected in Ukraine that spanned the same genomic region (HXB2 positions: 2,568–3,256) and which had available sampling dates and transmission group information. Only HIV-1 subtype B sequences with <5% ambiguous nucleotides were used. Duplicate sequences were removed using the ElimDupes tool from the Los Alamos website (https://www.hiv.lanl.gov). We used the REGAv3^19^ and COMET^20^ subtyping tools to confirm correct HIV subtype assignment.

After removing duplicates, the *Ukrainian dataset* contained 120 HIV-1 subtype B sequences with matching sociodemographic and epidemiological data (gender, transmission group, date of sampling, and city of sampling). We then downloaded the 10 closest reference sequences to the sequences in the Ukrainian dataset using the BLAST^21^ tool from the Los Alamos database website. After removing duplicates, these sequences and the Ukrainian dataset composed the *combined dataset* (*N* = 299 sequences). Information on reference sequences (country, date of sampling, and accession numbers) is provided in Supplementary Table S1.

#### Alignment

We codon-aligned the sequences using Clustal Omega^22^ and then manually edited the alignment using MEGA.^23^ We deleted 43 codon positions associated with drug resistance.^24^ The final alignment consisted of 614 nucleotides that correspond to nucleotide positions 2,568–3,256 of the HXB2 HIV reference sequence (*pol* gene). Mean pairwise genetic distances were calculated using MEGA.

#### Epidemiological data

The annual number of all newly registered HIV infections (all subtypes) by transmission group was available from the Ministry of Health website of Ukraine.^3^ Annual data were available starting from 1995.

#### Statistical analysis

To test for possible associations between sociodemographic and other characteristics, and the membership of sequences in the dominant circulating clades, we performed chi-square tests (for variables with expected cell values ≥5) and Fisher's exact tests (for variables with expected cell values ≤5). To account for multiple comparisons between different groups of the same variable, we used Bonferroni multiple test correction: only *p* values ≤ (.05/the number of categories in the variable) were considered significant.

### Phylogenetic analysis

#### Maximum likelihood phylogenies

We estimated maximum likelihood (ML) phylogenetic trees for the Ukrainian and the combined datasets using RAxML.^25^ We used a general time-reversible (GTR) nucleotide substitution model with gamma-distributed rate variation among sites. In the combined dataset, we identified two clades with >50 sequences, at least 75% of which were from Ukraine. Both clades exhibited SH-like statistical support values >0.80,^26^ which are considered statistically significant.^27^ We used RAxML to estimate ML trees for both clades after removing sequences that were not sampled in Ukraine. We used TempEst^28^ to evaluate root-to-tip divergence in the combined dataset and for the two Ukrainian clades.

We used BaTS^29^ to calculate the association index (AI) for phylogenies of the two Ukrainian clades. The AI is a statistic that describes the association between a phylogeny and traits at its tips (transmission groups in our case). BaTS also provides statistical support (*p* values) for the AI statistic by comparing values obtained from the posterior tree distribution with those obtained by randomly reassigning traits at the tips (100 times).

#### Time-calibrated phylogenies

We estimated evolutionary parameters and time-calibrated phylogenies using the BEAST, v1.8.4, software package.^30^ The combined dataset was used to estimate the time of the most recent common ancestor (TMRCA) of the two Ukrainian clades. To identify the most appropriate coalescent and molecular clock models, we used path-sampling^31^ and stepping stone^32^ marginal likelihood estimators. We assessed the fit of two molecular clock models (strict and lognormal relaxed) and three coalescent demographic models (constant population size, exponential growth, and Bayesian skyline). The Bayesian skyline model^33^ with a lognormal relaxed clock^34^ resulted in the best fit (Supplementary Table S2) and was used in further analyses. We used a GTR nucleotide substitution model with gamma-distributed rate variation among sites.

Markov Chain Monte Carlo (MCMC) analyses of the combined dataset were run for 200 million generations (with 10% burn-in). We then ran the Bayesian skyline analysis for 150 million generations (with 10% burn-in) under the same evolutionary model for the two Ukrainian clades separately to estimate fluctuations in effective population size (*N_e_*) for these two clades. Convergence of the MCMC sampler was inspected using Tracer.^35^

#### Estimates of effective reproductive number

The effective reproductive number (*R_e_*) of an epidemic is the average number of secondary infections attributable to an infected person. This value is often used to describe transmission dynamics in a population; *R_e_* > 1 means the epidemic is growing, while *R_e_* < 1 means the epidemic is declining. We used the phylodynamic birth–death model^36^ implemented in BEAST, v2.4.7,^37^ to estimate *R_e_* for the two Ukrainian clades.

We ran MCMC analyses for 150 million generations (10% burn-in). As before, we used a GTR nucleotide substitution model with gamma-distributed rate variation among sites. We applied a uniform prior for the date of origin of both clades, with a mean value of 50 years (lower bound = 0.0 and upper bound = 100.0). The sampling proportion was set to 0 before the first sampling date (2002 for both clades). After that a beta distribution prior was applied to the sampling proportion parameter (α = 1.0 and β = 10.0), with a constraint between 0.1% and 10%. We used a lognormal distribution prior for *R_e_* (mean = 1.0 and standard deviation = 1.25) and a normally distributed TMRCA prior for each of the two clades, using values of the TMRCA estimated in the previous analysis using the combined dataset (see Results section). We also assumed that the infectivity period, which is the period between contracting the virus and becoming uninfectious (being in treatment and virally suppressed, or dying), was, on average, 4 years for a patient in Ukraine; this parameter was fixed. To assess the robustness of our results to this assumption, we performed additional sensitivity analyses for this parameter by considering four additional values for the infectivity period: 1, 2, 6, and 8 years, which correspond to rates of becoming uninfectious of 1.0, 0.5, 0.167, and 0.125 per year.

## Results

**FIG. 1.**
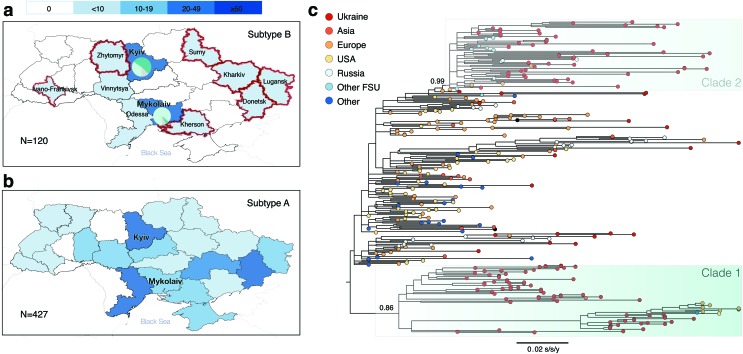
**(a)** The number and geographical distribution of HIV-1 subtype B sequences in the combined dataset. **(b)** The number and geographical distribution of HIV-1 subtype A sequences available from the Ukrainian Drug Resistance database in 2012–2015. Administrative units highlighted in *red* indicate newly sampled regions that were not represented in the Los Alamos HIV sequence database before this study. Pie charts for the Kyiv and Mykolaiv regions represent the proportions of sequences from these regions belonging to Clade 1 (*light green*), Clade 2 (*dark green*), or neither (*gray*). **(c)** The maximum likelihood phylogeny of the combined dataset with highlighted Clades 1 and 2. The *colors* at the tips represent the sampling regions. The *bar* at the *bottom* represents genetic distance. Values at the nodes for Clades 1 and 2 represent SH-like statistical support. Color images are available online.

Ukrainian HIV-1 subtype B sequences (*n* = 120) were obtained from patients belonging to four transmission groups: PWID (*n* = 26), MSM (*n* = 32), HET (non-PWID heterosexuals) (*n* = 54), and mother-to-child transmission (MTC, *n* = 8). Most of the Ukrainian sequences (75%, *n* = 90) were from male patients. Regarding their geographic distribution, 73 of the sequences (61%) came from Kyiv, 29 (24%) from Mykolaiv, and the remaining regions (*N* = 9) were represented by ≤5 sequences each (Table 1). The geographical distribution of the subtype B dataset used here is more representative than Ukrainian HIV-1 subtype B sequences available from the Los Alamos database (Fig. 1a), but is less extensive than that of the dominant HIV-1 subtype A in Ukraine (Fig. 1b).

**Table 1.** Sociodemographic Characteristics of the Ukrainian Sequences

*Full dataset*			*Clade 1*		*Clade 2*		*Chi-square/Fisher's exact test: Clade 1 vs. Clade 2*	*Not in Clade 1 or Clade 2*	*Chi-square/Fisher's exact test: “In the clades” vs. “Not”*	
*Variable*	N	*%*	*N*	*%*	N	*%*	p	N	*%*	p
All sequences										
Sampling region										
Ukraine	120	40.1	53	81.5	53	74.7	.409	14	8.6	**<.001**
Eastern Europe/Central Asia	32	10.7	0	0	15	21.1	**<.001**	17	10.4	0.87
World	147	49.2	12	18.5	3	4.2	**.012**	132	81.0	**<.001**
Total	299	100.0	65	100.0	71	100.0	—	163	100.0	—
Only Ukrainian sequences										
Gender										
Male	90	75.0	38	71.7	43	81.1	.253	9	64.3	.32
Female	30	25.0	15	28.3	10	18.9	.253	5	35.7	.32
Risk group										
HET	54	45.0	21	39.6	23	43.4	.693	10	71.4	.03
PWID	26	21.7	13	24.5	11	20.7	.642	2	14.3	.732
MTC	8	6.7	7	13.2	1	1.9	.06	0	0	.594
MSM	32	26.6	12	22.7	18	34.0	.176	2	14.3	.667
Region										
Kyiv	73	60.8	25	47.2	39	73.5	.005	9	64.3	.78
Mykolaiv	29	24.2	25	47.2	2	3.8	**<.001**	2	14.4	.514
Vinnytsya	5	4.2	—	—	4	7.5	.118	1	7.1	.468
Odessa	3	2.5	2	3.7	1	1.9	1	0	0	1
Dnipro	2	1.7	—	—	1	1.9	1	1	7.1	.221
Kharkiv	2	1.7	—	—	2	3.8	.495	0	0	1
Sumy	2	1.7	—	—	2	3.8	.495	0	0	1
Kherson	1	0.8	—	—	—	—	1	1	7.1	.117
Ivano-Frankivsk	1	0.8	—	—	1	1.9	1	0	0	1
Lugansk	1	0.8	1	1.9	—	—	1	0	0	1
Zhytomyr	1	0.8	—	—	1	1.9	1	0	0	1
Data source										
Ukrainian HIV drug resistance database	46	38.3	24	45.3	14	26.4	.04	8	57.2	.12
CASCADE	65	54.2	24	45.3	36	67.9	**.003**	5	35.7	.63
Los Alamos database	9	7.5	5	9.4	3	5.7	.716	1	7.1	1
Total	120	100.0	53	100.0	53	100.0	—	14	100.0	—

Values in *bold* indicate statistically significant *p*-values accounting for Bonferroni correction for multiple comparisons.

HET, heterosexuals; MSM, men who have sex with men; MTC, mother-to-child transmission; PWID, people who inject drugs.

Figure 1c shows the ML phylogenetic tree estimated from the combined dataset. This phylogeny reveals two clades that mostly contained Ukrainian sequences (≥75%): Clades 1 and 2, with SH-like support values of 0.99 and 0.86, respectively. Clade 1 contains 65 sequences (53 sequences from Ukraine, 4 from Finland, 3 from Germany, and 1 sequence each from United States, United Kingdom, Kenya, Belgium, and Japan). Clade 2 contains 71 sequences (53 sequences from Ukraine, 4 from Russia, 4 from Georgia, 2 each from Azerbaijan, Czech Republic, and Luxembourg, and 1 each from Belarus, Uzbekistan, Slovenia, and Italy).

A comparison of sociodemographic characteristics of Clades 1 and 2 is presented in Table 1. Clade 1 contains more sequences from Mykolaiv (Fisher's exact test *p*-value <.001). Sequences from Eastern Europe/Central Asia were more prevalent in Clade 2 (Fisher's exact test *p*-value <.001). There was no significant difference between the two clades in terms of transmission group or gender composition. Clade 2 had more CASCADE sequences (chi-square test *p*-value = .003) than Clade 1. We also compared sociodemographic characteristics of the sequences appearing in one of the two clades versus those that did not. Sequences in the two clades were more likely to be Ukrainian (chi-square test *p*-value <.001) and were less likely to be sampled in other parts of the world than Eastern Europe and Central Asia (Fisher's exact test *p*-value <.001).

The AI analysis showed transmission group admixture for both clades: AI values for Clades 1 and 2 are 4.32 (*p*-value .32) and 4.45 (*p*-value .60), respectively. The mean pairwise genetic distance was 2.9% in Clade 1 and 1.8% in Clade 2 (*t*-test *p*-value <.001). Since Clade 2 had lower pairwise genetic diversity, and included sequences from other FSU countries, this clade can be identified as the B_FSU_ strain, previously reported in the literature as originating from Mykolaiv.^7,14^

A regression of sampling time against root-to-tip genetic divergence showed a strong temporal signal in the combined dataset (correlation coefficient = 0.61), but weaker signal when the two Ukrainian clades were analyzed separately (correlation coefficients 0.3 and 0.27 for Clade 1 and Clade 2, respectively) (Supplementary Fig. S1). Therefore, we first ran a BEAST analysis on the combined dataset and estimated TMRCAs of Clades 1 and 2 to be 1971 [95% highest posterior density (HPD) 1956–1978] and 1973 (95% HPD 1965–1978), respectively. These dates were then used as priors on the TMRCA parameter for all subsequent Bayesian phylogenetic analyses.

**FIG. 2.**
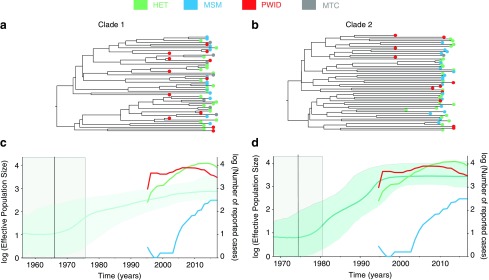
**(a, b)** Molecular clock trees for Clades 1 and 2, respectively. The *colors* at the tree tips represent patients' risk group. **(c, d)** Bayesian skyline plots for Clades 1 and 2, respectively. The *shaded green* area highlights the 95% HPD credible region. The *black vertical lines* and the *shaded gray area* represent the TMRCA and its 95% HPD credible region. The *colored lines* with the *right y*-axis represent the log number of reported HIV cases per risk group. HPD, highest posterior density; TMRCA, time to most recent common ancestor. Color images are available online.

The molecular clock trees of the two clades are presented in Figure 2a and b. When we analyzed the two clades separately, we obtained TMRCA estimates consistent with the analysis of the combined dataset (1967, 95% HPD 1956–1976, and 1975, 95% HPD 1968–1981, for Clade 1 and Clade 2, respectively) (Fig. 2c, d), as expected given the use of a strong TMRCA prior. For both clades, the values of the number of HIV infections in HET and PWID were almost a magnitude higher than the *N_e_* estimated by Bayesian skyline plots, and these values for MSM were magnitudes lower than *N_e_* estimates until the last 10 years (Fig. 2c, d). The *N_e_* of Clade 1 steadily grew over two orders of magnitude until 2017. The *N_e_* of Clade 2 grew ∼2.5 orders of magnitude within the same period; however, this clade appears to have experienced more rapid growth between 1980s and late 1990s. The overall number of reported HIV cases per year in HET and PWID, but not MSM, agree well with the estimated *N_e_* curves for Clade 2 (Fig. 2d).

**FIG. 3.**
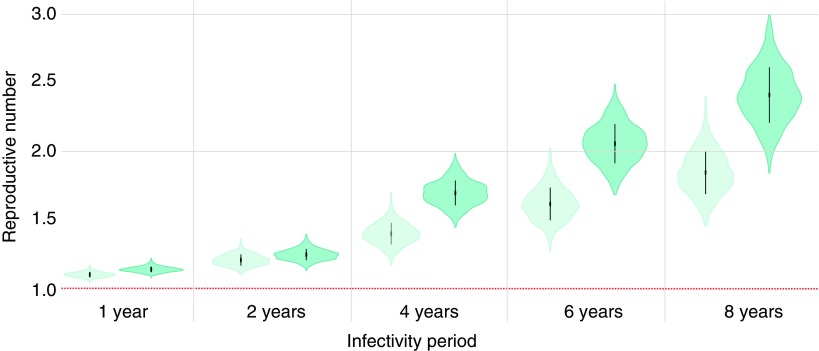
Estimates of the effective reproductive number (*R_e_*) for each clade, assuming different durations of infectivity. The *horizontal red dotted line* represents the epidemiological threshold (*R_e_* = 1). *Vertical lines* within violin plots reflect the second quartile of the *R_e_* distribution. Color images are available online.

We estimated *R_e_* for Clades 1 and 2 to be 1.42 (95% HPD 1.26–1.56) and 1.69 (95% HPD 1.49–1.84), respectively, under the assumption that the infectivity period is 4 years (Fig. 3). When we performed additional sensitivity analyses for the *R_e_* parameter, we found that (as expected) estimated values of *R_e_* increase as the infectivity period increases. Estimated *R_e_* remained between 1 and 2 if the infectivity period is assumed to be ≤4 years. If the infectivity period is considered to be >4 years, then *R_e_* gradually increases for both clades, with a particularly high *R_e_* for Clade 2 (*R_e_* = 2.42, 95% HPD 2.04–2.8) if we assume an infectivity period of 8 years. Importantly, the *R_e_* of Clade 2 was consistently higher than that of Clade 1 regardless of the specific value of the infectivity period used.

## Discussion

HIV-1 subtype A has dominated the epidemic in Ukraine and other FSU countries since the mid-1990s.^38^ The molecular epidemiology of subtype A in Ukraine has been well described in the late 1990s, when this HIV subtype was discovered in the country,^10,13^ and in recent years.^8,11,17^ However, very few studies were published between 2000 and 2015 even though the number of new HIV cases continued to grow rapidly in the early to late 2000s.^3,39,40^ The molecular epidemiology of HIV-1 subtype B in Ukraine has been mostly neglected, primarily due to its lower prevalence. In this study, we characterize the current molecular epidemiology of HIV-1 subtype B in Ukraine, including its transmission group composition and virus transmission dynamics.

Previous reports have suggested that the HIV-1 subtype B epidemic in Ukraine started with the introduction of this subtype into the drug-injecting community of Mykolaiv in 1990s.^7,41^ In 2001–2002, HIV-1 subtype B was mostly prevalent in Mykolaiv (*N* = 22), followed by Kyiv (*N* = 18), and few cases reported in Odessa.^7^ Two HIV-1 subtype B lineages were found circulating in Kyiv,^7^ which is consistent with our findings here. However, the geographic distribution of HIV-1 subtype B sequences in our study is quite different: the majority of HIV-1 subtype B sequences come from Kyiv (61% of the Ukrainian dataset) or other regions (15%). This could be explained by a potential selection bias in the CASCADE data,^17^ sampled exclusively in Kyiv. Among the available 46 HIV-1 subtype B sequences in the Ukrainian HIV Drug Resistance database, 24 (52%) originate from Mykolaiv, followed by small numbers of sequences from 9 other regions across Ukraine.

We find that Clade 2 (assigned as B_FSU_) is now found mostly in HET in Kyiv, even though it was previously identified predominantly in Mykolaiv's PWID population.^7,15^ The estimated effective population size of Clade 2 (B_FSU_) is congruent with the yearly number of HIV cases in both PWID and HET in Ukraine^3^ (Fig. 2d).

Overall, our analysis indicates a lack of transmission group structure for both clades, suggesting frequent viral mixing among transmission groups. Importantly, by analyzing the largest dataset of subtype B from Ukraine to date, we show that Ukrainian HIV-1 subtype B, B_FSU_ in particular, is no longer concentrated in selected regions (i.e., Mykolaiv and Kyiv) or in specific transmission groups. Similar observations of the expansion of B_FSU_ from PWID to other transmission groups have been made in Russia where HIV-1 subtype B sequences isolated from MSM patients clustered with B_FSU_ sequences collected from PWID.^42^

We also estimated the average reproductive number *R_e_* for the two clades of HIV-1 subtype B circulating in Ukraine. We found that Clade 2 (B_FSU_) has a consistently higher *R_e_* than Clade 1 regardless of the infectivity period value used. The *R_e_* values estimated for both clades (1.42 and 1.69, assuming an infectivity period = 4 years) were above one, indicating epidemic growth, but much lower than the *R_e_* value previously estimated at the beginning of the HIV-1 subtype A epidemic in Ukraine in the 1990s (*R_e_* = 10 per drug injector, although it is likely to be lower now).^12^ This difference in epidemic potential could be related to differences in drug use practices between PWID in the two cities, where subtypes A and B were first discovered (Odessa and Mykolaiv for subtypes A and B, respectively), or other cultural dissimilarities between the two cities, as described before.^41^ While Odessa had a large international port and also a large number of tourists, Mykolaiv was more isolated with some access restrictions during the Soviet era due to its close connection to the Soviet navy.^41^ This could be one of the explanations why HIV-1 subtype A rapidly became dominant in Ukraine, while HIV-1 subtype B remains less common to this day.

Another explanation is the first-comer advantage, which hypothesizes that the first successfully established founder strain can significantly slow down the spread of other strains in the region by either inhibiting superinfection, due to stimulation of anti-HIV immune responses and exhaustion of the number of target cells, or by reducing the number of susceptible noninfected individuals.^43^ For example, analyses of HIV-1 subtypes A and D in Uganda suggest that subtype A would need to have 25% transmission advantage compared with the first comer, subtype D, to establish an epidemic.^43^ The TMRCA of Ukrainian HIV-1 subtype A was previously estimated to be around 1970 (95% HPD 1962–1976),^11^ which is similar to the TMRCAs estimated here for HIV-1 subtype B Clade 2 (1973, 95% HPD 1965–1978) and Clade 1 (1971, 95% HPD 1956–1978).

Thus, our data suggest the approximately simultaneous introduction of HIV-1 subtypes A and B into Ukraine. However, the smaller number of HIV-1 subtype B sequences in our analysis, compared with previous HIV-1 subtype A studies, is associated with comparatively large confidence intervals for the estimates here. Further studies with more sequence data should provide a better understanding of the possible effects of first-comer advantage, if any, of HIV-1 subtypes in Ukraine.

One of the limitations of this study is that the data came from three different sources with different sampling techniques and different data collection methods for self-reported transmission group information. In particular, CASCADE data were collected anonymously, while data at the AIDS centers were collected by doctors in a nonanonymous manner. In our sample, 75% of patients were male; interestingly, 67% of HET patients were also male. MSM are often stigmatized and, as a consequence, this transmission group tends to be underreported in many settings, including Western Europe.^44–46^ We cannot exclude the possibility that some patients may have incorrectly self-reported as belonging to the non-MSM group in Ukraine. Similarly, PWID might not have disclosed their drug-injecting practices to AIDS center clinicians, particularly due to the stigma against HIV and drug use in medical facilities in Ukraine.^47,48^ As a result, underreporting of MSM and PWID status could overestimate the true proportion of HET in our study and thereby make the Ukrainian HIV-1 subtype B epidemiological profile look different from that of predominantly MSM-driven subtype B epidemics in Russia^49^ and some European countries.^50^

Furthermore, our sample is likely to represent a very small proportion of existing HIV-1 subtype B infections in Ukraine (<1%). This is partly due to high rates of undiagnosed HIV infections in Ukraine, particularly in some transmission groups (such as MSM).^51^ As very few patients have access to drug resistance testing,^8^ viral genetic data from routine care are also very sparse.

We found that the epidemiological profile of the HIV-1 subtype B epidemic in Ukraine has recently changed. Specifically, HIV-1 subtype B is no longer limited to the PWID subpopulation and is spreading to multiple transmission groups throughout the country. As with HIV-1 subtype A in Ukraine, the subtype B epidemic appears to have generalized to HET, a much larger susceptible population, which could fuel the spread of HIV in Ukraine. Our data also suggest that HIV-1 subtype B in Ukraine is not concentrated in MSM, unlike the situation in Western Europe. Although the Ukrainian HIV-1 subtype B epidemic appears to be growing comparatively slowly, and is characterized by a lower *R_e_*, its increasing range across locations and transmission groups highlights the need for better surveillance, linkage to care, antiretroviral treatment, and monitoring across the country.

## Ethical Approval

HIV drug resistance testing was performed at the L.V. Gromashevskij Institute of Epidemiology and Infectious Diseases of National Academy of Sciences of Ukraine following all Ukrainian legal and ethical requirements. Fully anonymized genetic sequence data were transferred to Oxford through a Material Transfer Agreement for molecular evolutionary analyses.

Ethical approval for CASCADE was given by the ethics committee of the L.V. Gromashevskij Institute of Epidemiology and Infectious Diseases of National Academy of Sciences of Ukraine and the ethics committee of University College London (UCL).

## Supplementary Material

Supplemental data

Supplemental data
