# Supplementary material for: The Changing Epidemiological Profile of HIV-1 Subtype B Epidemic in Ukraine
Source: AIDS Res Hum Retroviruses. 2019 Jan 31;35(2):155–63. doi: 10.1089/aid.2018.0167 (PMC6360399; doi:10.1089/aid.2018.0167)
Supplement: Supplemental data [file Supp_Fig1.pdf]

Supplementary Data

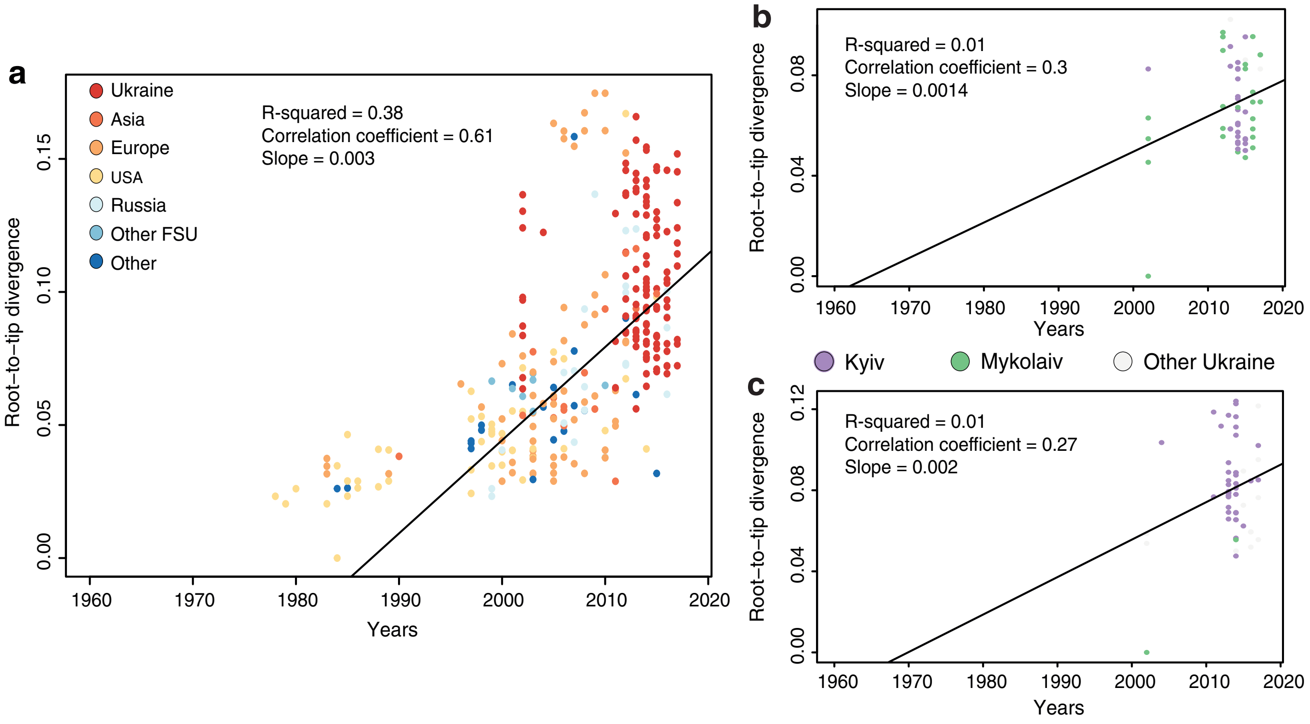

SUPPLEMENTARY FIG. S1. Root-to-tip divergence plots estimated using TempEst. **(a)** Combined dataset; **(b)** Clade 1; and **(c)** Clade 2.
